# Determining drug dose in the era of targeted therapies: playing it (un)safe?

**DOI:** 10.1038/s41408-022-00720-7

**Published:** 2022-08-23

**Authors:** Sigrid S. Skånland, Geir E. Tjønnfjord

**Affiliations:** 1 Department of Cancer Immunology, Institute for Cancer Research, Oslo University Hospital, Oslo, Norway; 2 The K. G. Jebsen Centre for B Cell Malignancies, Institute for Clinical Medicine, University of Oslo, Oslo, Norway; 3 Department of Haematology, Oslo University Hospital, Oslo, Norway

**Keywords:** Targeted therapies, Targeted therapies

## Abstract

Targeted therapies against phosphatidylinositol 3-kinase (PI3K), Bruton’s tyrosine kinase (BTK), and B-cell lymphoma-2 (BCL-2) are approved for chronic lymphocytic leukemia (CLL). Since approval of the first-in-class drugs, next-generation agents have become available and are continuously under development. While these therapies act on well-characterized molecular targets, this knowledge is only to some extent taken into consideration when determining their dose in phase I trials. For example, BTK occupancy has been assessed in dose-finding studies of various BTK inhibitors, but the minimum doses that result in full BTK occupancy were not determined. Although targeted agents have a different dose–response relationship than cytotoxic agents, which are more effective near the maximum tolerated dose, the traditional 3 + 3 toxicity-driven trial design remains heavily used in the era of targeted therapies. If pharmacodynamic biomarkers were more stringently used to guide dose selection, the recommended phase II dose would likely be lower as compared to the toxicity-driven selection. Reduced drug doses may lower toxicity, which in some cases is severe for these agents, and are supported by retrospective studies demonstrating non-inferior outcomes for patients with clinically indicated dose reductions. Here, we review strategies that were used for dose selection in phase I studies of currently approved and select investigational targeted therapies in CLL, and discuss how our initial clinical experience with targeted therapies have pointed to dose reductions, intermittent dosing, and drug combinations as strategies to overcome treatment intolerance and resistance.

## Phase I trial designs in the era of targeted therapies

With the introduction of rationally designed molecular cancer therapeutics, our understanding of drug discovery in cancer has undertaken a paradigm shift [1]. The aim of drug development strategies is to achieve maximal biological effect on the drug target, which will translate into therapeutic efficacy. As a result of this, a significant need has emerged for molecular biomarkers that precisely assess the underlying mechanisms of action and pharmacodynamic effects of the drug. Incorporation of such pharmacodynamic biomarkers in clinical trials may allow (i) proof of mechanism, i.e., evidence that the drug hits the intended target, (ii) proof of concept, i.e., evidence that hitting the target alters the biology of the tumor, (iii) determining optimal biological dosing, and (iv) understanding of response/resistance mechanisms [2].

**Fig. 1:**
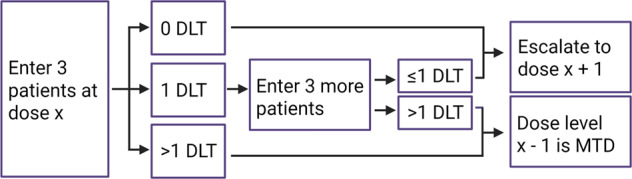
The 3 + 3 phase I trial design. DLT dose-limiting toxicity, MTD maximum tolerated dose.

In the traditional 3 + 3 phase I trial design [3], initially used to study cytotoxic agents, dose-limiting toxicity (DLT), rather than pharmacodynamic biomarkers, is used to guide dose escalation (Fig. 1). Three patients are first enrolled to a specified dose cohort. In the absence of any DLT, three additional patients are enrolled to a higher dose cohort. If one patient in the cohort develops a DLT, three more patients are enrolled to the same dose cohort. If no additional patients develop a DLT, that dose is defined as the maximum tolerated dose (MTD). If two or more of the six patients develop DLT, the MTD has been exceeded (Fig. 1). Notably, the MTD is determined already in the first cycle of therapy. Molecularly targeted therapies require longer treatment regimens than cytotoxic agents, and treatment emergent toxicities may appear later in the treatment course. While alternative phase I designs have been proposed for novel agents (Box 1) [4–6], the 3 + 3 design is still commonly used in chronic lymphocytic leukemia (CLL) trials (Table 1).

**Table 1:** Select phase I trials with targeted therapies in CLL.

Agent	Target	Clinicaltrials.gov identifier, reference	Dose-escalation design	Doses studied	Recommended phase II dose	Rationale for phase II dose recommendation	Comments
Ibrutinib	BTK	[12]	3 + 3	1.25, 2.5, 5, 8.3 or 12.5 mg/kg/day (intermittent schedule); 8.3 mg/kg/day or 560 mg/day (continuous schedule)	Continuous (12.5 mg/kg/day)	Dose three levels above the lowest dose which resulted in full BTK occupancy. Intermittent dosing led to reversal of treatment-related lymphocytosis	
Ibrutinib	BTK	NCT01105247 [19]	3 + 3	420 or 840 mg/day	420 mg/day	Full BTK occupancy and similar overall response rate with both doses	Lowest dose resulting in full BTK occupancy was not identified
Acalabrutinib	BTK	NCT02029443 [21]	3 + 3	100, 175, 250, or 400 mg/day	100 mg twice daily	Full BTK occupancy with 100 mg/day. Short half-life (1 h) and low toxicity allowed for twice-daily regimen	Lowest dose resulting in full BTK occupancy was not identified
Zanubrutinib	BTK	NCT02343120 [25]	3 + 3	40, 80, 160, or 320 mg once daily or 160 mg twice daily	160 mg twice daily	BTK occupancy was more frequent with the 160 mg twice daily regimen than with the 320 mg once daily administration	Only one dose (160 mg) was tested in the twice-daily regimen
Pirtobrutinib	BTK	NCT03740529 [26]	3 + 3	25, 50, 100, 150, 200, 250, or 300 mg/day	200 mg/day	Dose corresponding to unbound pirtobrutinib trough steady-state exposure with BTK plasma concentrations corresponding to 96% target inhibition	Estimated BTK inhibition was only reported for the recommended phase II dose (200 mg/day)
Idelalisib	PI3K	NCT00710528, NCT01090414 [27]	3 + 3	300 mg once daily or 50, 100, 150, 200, or 350 mg twice daily	150 mg twice daily	Patients treated with ≥150 mg twice daily had a longer PFS than those treated with a lower dose	
Duvelisib	PI3K	NCT01476657 [28]	3 + 3	8, 15, 25, 35, 50, 60, 75, or 100 mg twice daily	25 mg twice daily	Maximal pAKT and Ki67 effects and clinically meaningful activity	Once daily regimens were not studied
Umbralisib	PI3K	NCT01767766 [32]	3 + 3	50, 100, 200, 400, 800, 1200, or 1800 mg once daily (fasting), or 200, 400, 800, 1000, 1200, 1800 mg once daily (fed)	800 mg/day	Decreases in tumor burden plateaued at plasma concentrations above the minimum target exposure of 3000 ng/mL obtained with 800 mg/day	
Venetoclax	BCL-2	NCT01328626 [35]	3 + 3	Ramp-up from 50 mg to 150, 200, 300, 400, 600, 800, or 1200 mg/day	400 mg/day	Balance of overall response and safety data	

*BCL-2* B-cell lymphoma-2, *BTK* Bruton’s tyrosine kinase, *PI3K* phosphatidylinositol 3-kinase.

The task force on Methodology for the Development of Innovative Cancer Therapies (MDICT) was established in 2006 to provide practical guidance on the development of anticancer targeted agents. In 2008, they suggested that MTD and pharmacokinetics are reasonable phase I endpoints to determine the dose of targeted agents [7]. The rationale for this recommendation was not explicitly formulated, but appears to be based on a review of 57 phase I trials on 31 targeted agents demonstrating that toxicity was the most common determinant for halting dose escalation and defining dose recommendation for further studies [7, 8]. The MDICT additionally recommended to confirm that the selected dose affects the molecular target as predicted. These recommendations have to varying degree been followed in dose-finding studies of targeted therapies in CLL, as discussed below.

Box 1 Alternatives to the 3 + 3 phase I trial design in the era of targeted therapies The 3 + 3 trial design (Fig. 1) was initially developed to determine the dose of cytotoxic agents, which are more effective near the maximum tolerated dose (MTD). Molecularly targeted therapies require longer treatment regimens than cytotoxic agents, and treatment emergent toxicities may appear later in the treatment course. To capture these features when determining the dose of novel agents, it has been suggested to improve the 3 + 3 trial design by (i) using toxicity-adjusted dose escalation rather than predetermined schemes, (ii) increasing the number of patients, and (iii) extending the dose-limiting toxicity (DLT) window beyond the first treatment cycle [4].

In addition to the rule-based 3 + 3 trial design, model-based and model-assisted designs such as the continual reassessment method (CRM) have evolved [4]. The model-based designs build on the principle of constantly updating the estimated toxicity rates based on available safety data [9]. They use a prespecified statistical model, and not a predetermined algorithm. This means that the dose the next patient will be treated with is unknown unless information about the dose of the previous patient can be integrated in the model. According to the U.S. Food and Drug Administration (FDA), the model-based designs are more likely to recommend the correct MTD and dose more patients appropriately [10]. However, a review of 1712 dose-finding studies published between 2008–2014 showed that 92.9% used a rule-based design while only 5.4% used a model-based or another novel design [11]. The limited use of the model-based designs may be due to their complex methodology which requires high statistical expertise in the clinical community. Optimization of the familiar 3 + 3 design is therefore more likely to be embraced by the clinical investigators and may result in improved dose determination of novel targeted therapies and better patient care.

## Dose-finding studies of targeted therapies in CLL

We reviewed the strategies that were used for dose selection in phase I studies of currently approved and select investigational targeted therapies in CLL (Table 1). From each study, we collected data on starting dose, method of dose escalation, and determination of recommended phase II dose.

### BTK inhibitors

In a phase I open-label, dose-escalation study of the first-in-class covalent Bruton’s tyrosine kinase inhibitor (BTKi) ibrutinib in relapsed/refractory (R/R) B-cell non-Hodgkin lymphoma (NHL) and CLL, the level of ibrutinib occupancy of BTK was used as a biomarker when determining the recommended phase II dose [12]. This was based on findings from a study in dogs that demonstrated correlation between treatment efficacy and BTK occupancy [13]. On the phase I trial, patients received ibrutinib orally once a day (omne in die; OD) at 1.25, 2.5, 5, 8.3, or 12.5 mg/kg on a 28 days on, 7 days off schedule, or continuously at 8.3 mg/kg or 560 mg/day (Table 1) [12]. Dose-escalation proceeded after assessment of DLT at end of cycle one (35 days). The MTD was defined as the dose where ≥33% of the patients experienced a DLT or at three dose levels above the lowest dose which resulted in full BTK occupancy if no DLT was observed. The rationale for increasing the dose with three levels is questionable as this by default means that the highest or second-highest dose would be selected since only five doses were tested. On the study, the MTD of ibrutinib was not reached, and only two DLTs were reported for the full cohort of 56 patients. A BTK occupancy >95% was achieved for all dose levels between 2.5 and 12.5 mg/kg/day with similar response rates. Although not explicitly stated, this indicates that the recommended phase II dose was 12.5 mg/kg/day. A continuous schedule was recommended since CLL patients experienced transient reversal of treatment-related lymphocytosis during the 7 days off on the intermittent dosing schedule (Table 1) [12]. Lymphocytosis is a common effect of ibrutinib and other B-cell receptor inhibitors and represents lymphocyte egress from nodal compartments [14–18]. The lymphocytosis is transient, and it is not associated with adverse events, inferior progression free survival (PFS), or disease progression [16–18].

In a subsequent phase Ib-II study of ibrutinib treatment of 85 patients with R/R CLL, 51 patients received 420 mg, and 34 patients received 840 mg OD (Table 1) [19]. This corresponds to 6 mg/kg and 12 mg/kg in a 70 kg patient, respectively. Full occupancy of BTK was observed at both doses, and the overall response rate was the same for both groups [19]. Based on this, the 420 mg dose was suggested for relapsed CLL (Table 1). This dose is lower than what was recommended in the initial phase I trial [12].

**Fig. 2:**
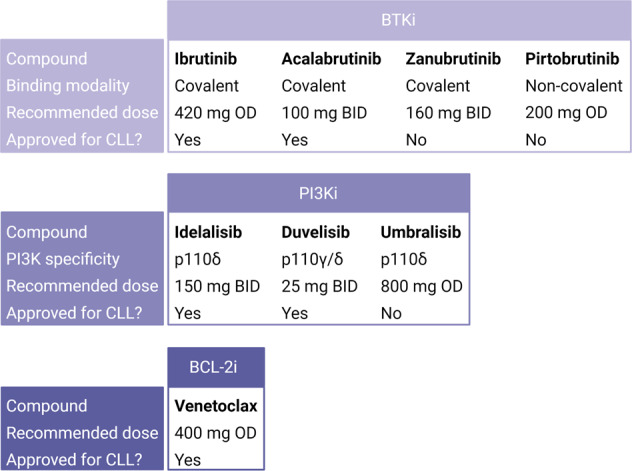
Approved and select investigational targeted therapies in CLL. BCL-2i B-cell lymphoma-2 inhibitor, BID bis in die (twice a day), BTKi Bruton’s tyrosine kinase inhibitor, CLL chronic lymphocytic leukemia, PI3Ki phosphatidylinositol 3-kinase inhibitor, OD omne in die (once daily).

In 2014, the U.S. Food and Drug Administration (FDA) granted ibrutinib accelerated approval for patients with CLL who have received at least one prior therapy. In 2016, ibrutinib was approved as frontline treatment for CLL, based on the RESONATE-2 study where it was compared to chlorambucil [20]. In 2019, the FDA approved the use of ibrutinib in combination with obinutuzumab (anti-CD20 antibody) for the treatment of adult patients with previously untreated CLL and in 2020 expanded the indication to include its combination with rituximab (anti-CD20 antibody) for frontline treatment of CLL. It is also approved by the European Medicines Agency (EMA). The recommended ibrutinib dose is 420 mg taken orally OD (Fig. 2).

Acalabrutinib is a second-generation covalent BTKi. It is more selective than ibrutinib with less off-target effects resulting in less adverse events (AEs). In a phase I–II study of acalabrutinib in 61 relapsed CLL patients, the agent was administered at 100–400 mg OD in the dose-escalation part of the study (Table 1) [21]. Complete BTK occupancy (99–100%) was observed already with the lowest dose. The half-life of acalabrutinib was only 1 h whereas the ibrutinib half-life is 4–13 h [22]. This characteristic, combined with the low toxicity of acalabrutinib, allowed for twice-daily dosing in the phase II part of the study [21].

The FDA approved acalabrutinib for the treatment of CLL in 2019, based on the ELEVATE-TN and ASCEND studies [23, 24]. It was approved by the EMA as a monotherapy for previously treated CLL in 2020. The recommended dose is 100 mg orally every 12 h (Fig. 2).

Zanubrutinib is another selective, covalent BTKi. In a phase I study of zanubrutinib in R/R B-cell malignancies, patients received the agent at 40, 80, 160, or 320 mg OD or at 160 mg twice daily (bis in die: BID) (Table 1) [25]. Median BTK occupancy was >95% for all doses, but sustained BTK occupancy was more frequent with the 160 mg BID regimen than with the 320 mg OD administration [25]. This was the rationale for recommending 160 mg BID as the phase II dose (Table 1). It would have been of interest to expand the study to also include 40 or 80 mg BID schedules since these doses, when administered OD, performed as well as the 160 mg OD regimen. Zanubrutinib is approved by the FDA for treatment of mantle cell lymphoma (2019) and Waldenström’s macroglobulinemia (2021), but not yet for CLL (Fig. 2). Both 160 mg BID and 360 mg OD dosings are approved by the FDA.

Pirtobrutinib is an investigational first-generation, non-covalent BTKi, which is effective also in BTK C481-mutant CLL (Fig. 2) [26]. In a phase I study of pirtobrutinib in 323 patients with B-cell malignancies, the agent was administered at 25, 50, 100, 150, 200, 250, or 300 mg OD (Table 1) [26]. No DLTs were observed. The recommended phase II dose was set to 200 mg/day based on an estimated target inhibition of 96% [26]. Target inhibition achieved with the other doses was not reported.

### PI3K inhibitors

Idelalisib is a phosphatidylinositol 3-kinase inhibitor (PI3Ki), more specifically it blocks p110δ. In a phase I study, 54 patients with R/R CLL received idelalisib 300 mg OD or 50, 100, 150, 200, or 350 mg BID (Table 1) [27]. OD dosing did not maintain continuous plasma exposure to the same level as BID dosing did. Further, patients treated with ≥150 mg BID had a longer PFS than those treated with a lower dose (32 months versus 7 months, respectively) [27]. Based on this, 150 mg BID was recommended as the phase II dose. Idelalisib was EMA and FDA approved for CLL in 2014. The recommended dose is 150 mg BID (Fig. 2).

Duvelisib is a next-generation, dual p110γ/δ PI3Ki. In a phase I dose-escalation study, 31 patients with advanced hematologic malignancies were treated with 8, 15, 25, 35, 50, 60, 75, or 100 mg duvelisib BID (Table 1) [28]. The half-life of duvelisib is 5.2–10.9 h, similar to that of ibrutinib [22]. Since the long half-life of ibrutinib was used as an argument for not testing a BID regimen for that agent, one may ask why an OD regimen was not studied for duvelisib. MTD was determined to be 75 mg BID based on occurrence of DLT for 1/6 patients receiving 75 mg and for 2/2 patients receiving 100 mg duvelisib [28]. Inhibition of PI3K signaling (pAKT) and proliferation (Ki67) was not dose-dependent, and were highest at 25 mg. The study was expanded with 179 patients who received duvelisib at 25 or 75 mg BID. Grade ≥3 AEs and overall response rates were similar for the two cohorts [28]. Based on these findings, 25 mg BID was recommended as the phase II dose.

Duvelisib was approved by the FDA in 2018 for treatment of CLL patients who have received at least two prior therapies. The approval was based on the DUO study where duvelisib was compared to the anti-CD20 antibody ofatumumab [29]. Approval by the EMA followed in 2021. The recommended dose is 25 mg BID (Fig. 2). Both idelalisib and duvelisib have a severe toxicity profile which have led to addition of black box warnings for both agents [30, 31]. Furthermore, the developers of these agents recently voluntarily withdrew the accelerated approvals for follicular lymphoma (FL) due to the inability to complete the confirmatory trial.

Umbralisib is a p110δ selective PI3Ki. In a phase I study of umbralisib in patients with R/R CLL and lymphoma, patients received umbralisib in a fasting state at 50, 100, 200, 400, 800, 1200, or 1800 mg OD (Table 1) [32]. Additional cohorts then received umbralisib in a fed state at 200, 400, 800, 1000, 1200, or 1800 mg OD (micronised formulation) [32]. The half-life of umbralisib was more than 100 h. Two DLTs were reported in patients who received 1800 mg/day of the micronised formulation. The MTD was therefore determined to be 1200 mg/day. The plasma concentration of umbralisib remained above the minimum target exposure of 3000 ng/mL (5.25 µM) when administered at 800 or 1200 mg. At plasma concentrations exceeding 3000 ng/mL, the decreases in tumor burden plateaued. Based on this finding, 800 mg/day was recommended as the phase II dose (Table 1) [32].

Umbralisib received an FDA fast-track approval status for CLL in combination with the anti-CD20 antibody ublituximab in 2020, and was FDA approved for FL and marginal zone lymphoma (MZL) in 2021. The recommended dose is 800 mg OD (Fig. 2). However, analyses of six randomized controlled trials with PI3Ki in indolent NHL or CLL led to concerns about inferior overall survival in the PI3Ki arms and subsequent voluntary withdrawal of umbralisib from the market for approved indications and of the application for the combination of umbralisib plus ublituximab for CLL and SLL [33, 34]. A comment by the FDA emphasized the need for careful dose selection for PI3Ki, preferably in randomized trials [33].

### Venetoclax

Venetoclax is a B-cell lymphoma-2 (BCL-2) antagonist. In a phase I study of venetoclax, patients first received a test dose of 20 mg or 50 mg to test for occurrence of tumor lysis syndrome. The patients then received venetoclax following a 3-week ramp-up scheme to final doses of 150, 200, 300, 400, 600, 800, or 1200 mg OD (Table 1) [35]. The expansion cohort received a final dose of 400 mg after a 5-week ramp-up starting at 20 mg OD. The half-life of venetoclax after a 50 mg dose was ~19 h. Venetoclax was active at all studied doses. The PFS at 15 months were 58%, 69%, and 77% for patients receiving <400, 400, and >400 mg, respectively [35]. According to the authors of the study, the recommended phase II dose of 400 mg OD was determined based on response and safety data (Table 1), but further details were not provided [35].

In 2016, venetoclax was approved by the FDA for previously treated CLL patients with del(17p). Based on the MURANO study [36], a randomized phase III trial comparing venetoclax plus rituximab with bendamustine plus rituximab in patients with R/R CLL, the FDA in 2018 approved venetoclax for CLL patients, with or without del(17p), who have received at least one prior therapy. The CLL14 study compared venetoclax plus obinutuzumab with chlorambucil plus obinutuzumab in previously untreated CLL patients [37]. After a median follow-up of 28.1 months, the estimated PFS at 24 months was 88.2% in the venetoclax arm and 64.1% in the obinutuzumab arm [37]. Based on this study, the FDA granted a general approval of venetoclax for all patients with CLL in 2019. The recommended dosing is a 5-week ramp-up from 20 mg to 400 mg OD (Fig. 2).

## Lower doses of targeted therapies do not compromise outcome

An ethical concern of the 3 + 3 phase I trial design (Fig. 1) has been that several patients may be treated with a sub-optimal dose. However, a systematic analysis of 683 patients treated with doses below, at, or above the MTD on 24 phase I trials showed that patients treated with lower doses of targeted therapies did not show worse outcome than other patients on the trials [38]. This finding suggests that targeted therapies have a different dose–response relationship than cytotoxic agents, which are more effective near the MTD.

A clinical pilot study investigated the pharmacokinetic and pharmacodynamic effects of reducing ibrutinib from the recommended dose of 420 mg/day via 280 mg/day to 140 mg/day over three 28-day cycles [39]. The study showed that BTK occupancy, inhibition of BTK downstream signaling, and autophosphorylation (Tyr223), as well as reductions of plasma chemokine CCL3 and CCL4 levels, were similar at the three dose levels [39], indicating that the currently recommended dose is superfluously high if the effects of ibrutinib are on target. In support of these findings, several retrospective studies have shown that clinically indicated reductions of ibrutinib dose do not compromise outcome in CLL [40–46]. Furthermore, ibrutinib dose intensity did not affect PFS in a prospective study of CLL with aberrant TP53 [47]. However, while ibrutinib dose intensity does not impact patient outcome, missed ibrutinib doses may [44, 48]. A retrospective study of the phase III RESONATE trial showed that median PFS was shorter in patients missing ≥8 consecutive days of ibrutinib compared to patients missing <8 days [48], and a retrospective study of 315 patients in UK and Ireland showed that patients with >14 days of ibrutinib discontinuation during the first year of treatment had reduced 1 year overall survival compared to the entire cohort (68.5% vs. 83.8%) [44]. Dose interruptions and dose modifications do not affect PFS of CLL patients on venetoclax [49].

## Lessons learned from PI3K inhibitor toxicity—intermittent dosing

PI3Ki are effective in CLL, but the serious toxicities associated with the first-generation inhibitors idelalisib and duvelisib have limited their use [50]. Prolonged exposure to this class of targeted therapies has been reported to increase the incidence of adverse events [51], making phase I studies with the traditional 3 + 3 design challenging since the dose is determined after the first treatment cycle. Alternative trial designs should therefore be considered for these agents (Box 1). Even so, lessons learned from our initial clinical experience with PI3Ki has allowed for development of more specific next-generation inhibitors and optimized treatment schedules. A strategy to overcome treatment toxicity is to change from continuous to intermittent dosing regimens. A retrospective study of idelalisib plus rituximab treatment of CLL demonstrated that treatment benefit extended far beyond treatment duration (median PFS 29.6 months, median treatment duration 11.9 months) [52]. This finding warrants studies of time-limited or intermittent dosing of idelalisib in prospective clinical trials. Such alternative dosing regimens are already established for the two PI3Ki copanlisib and zandelisib, and is under investigation for additional agents.

Zandelisib is a next-generation p110δ inhibitor with longer p110δ occupancy than idelalisib [53, 54]. A phase I study of zandelisib in healthy volunteers identified 60 mg OD as the recommended phase II dose based on high inhibition of basophil activation [54]. A phase Ib study of zandelisib in FL and CLL/SLL showed that the most common AEs had a delayed onset beyond cycle 2 [55]. These AEs could be reversed by treatment interruption. These findings motivated a phase I trial with intermittent dosing (7 days on/21 days off) after two continuous cycles [56]. The rationale for the time off was based on the time it took for the regulatory T cells to repopulate. Preliminary results indicate that the intermittent dosing maintains efficacy but reduces the rate of delayed grade 3 AEs [56]. The phase II TIDAL trial was designed to compare continuous and intermittent dosing regimens in patients with R/R FL, but has been revised to only study intermittent dosing (NCT03768505) [57]. The ongoing phase III COASTAL study is only studying an intermittent schedule for zandelisib (NCT04745832) in patients with relapsed indolent NHL [58]. In 2020, the FDA granted zandelisib fast-track designation for treatment of adult patients with R/R FL who have received at least 2 prior systemic therapies. Combination studies of zandelisib with rituximab, zanubrutinib or venetoclax in CLL are ongoing (NCT02914938, NCT05209308).

Parsaclisib is another next-generation p110δ inhibitor [59]. Intermittent dosing of this agent was studied in a phase I trial in patients with R/R B-cell malignancies [60]. Parsaclisib was administered at 20 mg OD for the first 9 weeks followed by 20 mg once weekly to decrease late onset AEs. This design was based on the comparative pharmacokinetic and pharmacodynamic simulation with the p110α/β/γ/δ inhibitor copanlisib [60, 61]. No treatment discontinuations were reported due to AEs in the intermittent dosing arm, while 13% of the patients on the continuous dosing arm discontinued treatment. High-grade AEs were also fewer in the intermittent dosing arm. The phase Ib/IIa topMIND trial is studying intermittent dosing of parsaclisib in combination with tafasitamab (anti-CD19 antibody) in R/R CLL (NCT04809467).

These studies suggest that intermittent dosing is a strategy to overcome intolerability to PI3Ki. This strategy is now studied also for duvelisib. In the phase II TEMPO trial (NCT03961672) on CLL/SLL, duvelisib is first administered continuously for three cycles, then on days 1–2, 8–9, 15–16, 22–23 of each cycle.

## Summary and outlook

Our review of dose-finding studies for targeted therapies in CLL demonstrates that the traditional 3 + 3 design is still heavily used for novel agents in CLL. As determination of DLT is of less relevance for novel agents than for cytotoxic agents, additional read-outs were considered in each trial (Table 1). However, it is only for BTKi that the direct effect on the drug target (BTK occupancy) is consistently studied across trials (Table 1). For PI3Ki it has proven more difficult to identify a molecular endpoint, such as reduced signaling downstream of PI3K. However, when an appropriate molecularly targeted endpoint is available, as is the case for BTKi, using this to guide dose selection is likely to indicate a lower dose than what would be obtained from a toxicity-driven design [62]. This is underscored by the finding that the minimum BTKi dose resulting in full BTK occupancy was either not identified (the lowest dose tested resulted in full BTK occupancy) or reported (only reported for the recommended phase II dose) in the trials reviewed here (Table 1). This suggests that there is still room to optimize the dose of these agents, which is in agreement with the many reports showing that clinically indicated reductions in ibrutinib dose do not compromise outcome in CLL [40–46, 48].

Combination regimens with targeted therapies are increasingly relevant in CLL as a strategy to deepen responses and overcome resistance [63]. This means that patients potentially will experience side effects from more than one agent. This should be taken into consideration when recommending phase II doses, as a lower drug dose may result in less toxicity. Drugs that are combined may exert synergy, which will amplify their individual contributions. This further justifies the use of lower drug doses. Ex vivo treatment of CLL cells with ibrutinib plus venetoclax demonstrated that drug synergy occur at doses that are much lower than the recommended treatment doses [64]. The CORAL study (NCT05209308), which investigates the combination of zandelisib with venetoclax and rituximab in R/R CLL, will include an initial phase I study of reduced venetoclax dose, demonstrating that the recommended doses of targeted agents are continuously evaluated.

In summary, the traditional toxicity-driven 3 + 3 phase I trial design is still dominating in the era of targeted therapies. While pharmacodynamics biomarkers were studied in most of the reviewed trials, these biomarkers did not weigh heavy when determining the phase II dose. If the pharmacodynamic biomarkers had been used more stringently to guide dose selection, the recommended dose would in the majority of cases be set lower than the currently recommended dose. As dose reductions can lower treatment-related and financial toxicity that patients and health care systems experience, and do not result in inferior outcomes, we believe it is overdue to let the targeted effects of targeted agents guide dose selection.
